# Ginkgo biloba extracts improve choroidal circulation leading to suppression of myopia in mice

**DOI:** 10.1038/s41598-023-30908-1

**Published:** 2023-03-07

**Authors:** Jing Hou, Kiwako Mori, Shin-ichi Ikeda, Heonuk Jeong, Hidemasa Torii, Kazuno Negishi, Toshihide Kurihara, Kazuo Tsubota

**Affiliations:** 1 Department of Ophthalmology, Keio University School of Medicine, 35 Shinanomachi, Shinjuku-ku, Tokyo, 160-8582 Japan; 2 Laboratory of Photobiology, Keio University School of Medicine, 35 Shinanomachi, Shinjuku-ku, Tokyo, 160-8582 Japan; 3 Tsubota Laboratory, Inc., 304 Toshin Shinanomachi-ekimae Bldg., 34 Shinanomachi Shinjuku-ku, Tokyo, 160-0016 Japan

**Keywords:** Refractive errors, Experimental models of disease, Preclinical research

## Abstract

Myopia is becoming more common across the world, necessitating the development of preventive methods. We investigated the activity of early growth response 1 (EGR-1) protein and discovered that Ginkgo biloba extracts (GBEs) activated EGR-1 in vitro. In vivo, C57BL/6 J mice were fed either normal or 0.0667% GBEs (200 mg/kg) mixed chow (n = 6 each), and myopia was induced with − 30 diopter (D) lenses from 3 to 6 weeks of age. Refraction and axial length were measured by an infrared photorefractor and an SD-OCT system, respectively. In lens-induced myopia mice, oral GBEs significantly improved refractive errors (− 9.92 ± 1.53 D vs. − 1.67 ± 3.51 D, *p* < 0.001) and axial elongation (0.22 ± 0.02 mm vs. 0.19 ± 0.02 mm, *p* < 0.05). To confirm the mechanism of GBEs in preventing myopia progression, the 3-week-old mice were divided into normally fed with either myopic-induced or non-myopic-induced groups and GBEs fed with either myopic-induced or non-myopic-induced groups (n = 10 each). Choroidal blood perfusion was measured with optical coherence tomography angiography (OCTA). In both non-myopic induced groups, compared to normal chow, oral GBEs significantly improved choroidal blood perfusion (8.48 ± 15.75%Area vs. 21.74 ± 10.54%Area, *p* < 0.05) and expression of *Egr-1* and *endothelial nitric oxide synthase (eNOS)* in the choroid. In both myopic-induced groups, compared to normal chow, oral GBEs also improved choroidal blood perfusion (− 9.82 ± 9.47%Area vs. 2.29 ± 11.84%Area, *p* < 0.05) and was positively correlated with the change in choroidal thickness. These findings suggest that GBEs may inhibit the progression of myopia by improving choroidal blood perfusion.

## Introduction

Myopia is a significant eye condition that affects people all over the world and half of the world's population is expected to be short-sighted by 2050 as the incidence of myopia increases year by year^1–3^. With the novel coronavirus outbreak and a series of lockdowns and home quarantine policies, restrictions on outdoor activities have revived the topic of myopia^4,5^. How to eliminate or reduce the development of myopia is a serious problem that cannot be ignored.

At present, there are various strategies to restrict the progression of myopia. In addition to the well-known pharmacological interventions (e.g., atropine, pirenzepine, 7-methylxanthine) and optical interventions (e.g., orthokeratology, peripheral defocus lenses)^6,7^, spending more time outdoors has also emerged as one of the safest and most important strategies to reduce the development of myopia^8–10^. The most important factor in increasing time spent outdoors is exposure to bright outdoor light^11^.

Outdoor light has a spectral composition that is dominated by short-wavelength visible components like blue and green rather than red^12^. Furthermore, blue light has been reported to inhibit myopia^13^. In our previous studies, violet light in the outdoor environment, which has a shorter wavelength than blue light, has been demonstrated to suppress the development of myopia in chick and murine myopia models and humans, and in addition, violet light exposure upregulates myopia suppressive gene *Egr-1* both in vitro and vivo^14,15^. *Egr-1* is a protein-coding gene that controls axial elongation and progression of myopia^16–18^. Moreover, *Egr-1* knockout mice exhibited longer axial length and a myopic refraction shift^19–21^. Realizing that *Egr-1* expression could be used as an evaluation gene for myopia suppression, we performed luciferase assays in the cultured cell line to screen 207 natural substances and organic chemicals and found that 75% or above the purity of crocetin-containing gardenia fruit extract A demonstrated the maximum activation of EGR-1^22^. Crocetin has been demonstrated to suppress the development of experimental myopia in mice and may have a suppressive effect on myopia progression in children^22,23^. Ginkgo biloba extracts (GBEs) were found to be the second strongest activator of EGR-1^22^. Therefore, it is reasonable to assume that GBEs, like crocetin, also have an inhibitory effect on the progression of myopia.

Ginkgo is a large tree with fan-shaped leaves. It is native to China, Japan, and Korea, but is also now grown in Europe and the United States. Traditional Chinese medicine has utilized the fruits and seeds of the Ginkgo biloba tree for over 5000 years, with recommendations for the treatment of asthma, cough, and enuresis^24,25^. A German pharmaceutical business created a ginkgo biloba extract called EGb 761 in 1964, and hundreds of research have explored ginkgo's effects in human and animal models since then^26^. After decades of persistent research, GBEs have been shown to exhibit a wide range of biological actions, including antioxidant^27^, antiviral^28^, anti-inflammation^29,30^, anti-tumor^31,32^, immunomodulation^33^, and hepatoprotective properties^34^. Remarkably, GBEs have also emerged as a potential treatment for glaucoma and other ischemic ocular diseases for their beneficial impact on blood circulation^35,36^. Increasing axial elongation in myopia results in thinning of retinal, choroidal, and scleral tissues, and interferes with ocular blood flow. It was found that in guinea pigs, choroidal thickness and choroidal blood perfusion were decreased during experimental myopia induction and then increased during recovery^37–39^. Consequently, we conducted a study designed to evaluate further whether oral GBEs administration can suppress the progression of experimental myopia in a murine model and determine if GBEs inhibit myopia by increasing choroidal blood perfusion.

## Results

### In a luciferase assay, GBEs generated a significant activation of EGR-1

**Figure 1:**
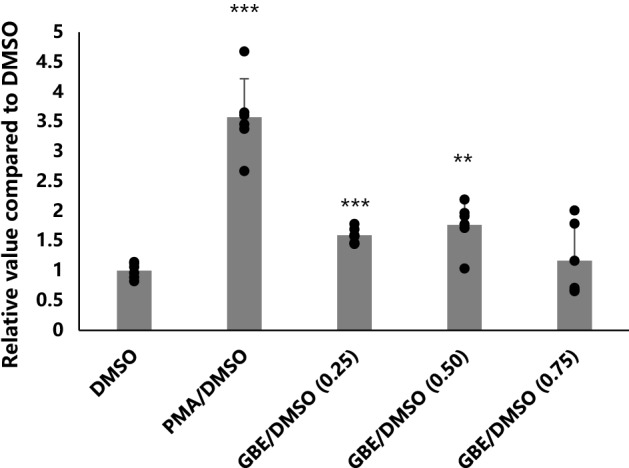
Effects of various GBEs concentrations on EGR-1 activation in a luciferase assay. GBEs were tested at three different doses, and both 0.25 mg/ml and 0.5 mg/ml concentrations of GBEs showed a statistically significant increase in EGR-1 activity in a luciferase assay (n = 6). **p < 0.01, ***p < 0.001. Bars represent mean + / − standard deviations. The one-way ANOVA was used for statistical analysis. EGR-1: early growth response protein 1; GBEs: Ginkgo biloba Extracts; DMSO: dimethyl sulfoxide; PMA: phorbol 12-myristate 13-acetate.

The effect of different concentrations of GBEs on the activity of EGR-1 was detected by human embryonic kidney adeno-associated virus EGR-1-Luc cell (HEK 293AAV EGR-1-Luc cell) lines, this cell line was obtained by a series of transfection and passages of HEK 293AAV cell line, which was purchased from Cell Biolabs, Inc. In this study, three concentrations, 0.25 mg/ml, 0.50 mg/ml, and 0.75 mg/ml of GBEs were used for EGR-1 activity assays, dimethyl sulfoxide (DMSO) without compounds was used as the negative control, and phorbol 12-myristate 13-acetate (PMA) was used as the positive control (n = 6 for each group). Values from the experimental group and the positive control group are shown as multiples compared with the negative control group. Compared with the negative control group, the positive control group showed a statistically significant increase in EGR-1 activity, the activation was 3.57 ± 0.65 folds (*p* < 0.001). For 0.25 mg/ml of GBEs, the EGR-1 activation was 1.59 ± 0.13 folds (*p* < 0.001), for 0.50 mg/ml of GBEs, the EGR-1 activation was 1.77 ± 0.40 folds (*p* < 0.01), and for 0.75 mg/ml of GBEs, the EGR-1 activation was 1.17 ± 0.60 folds (*p* = 0.520) (Fig. 1).

### Oral GBEs administration suppressed myopia progression in a lens-induced myopia model of mice

**Figure 2:**
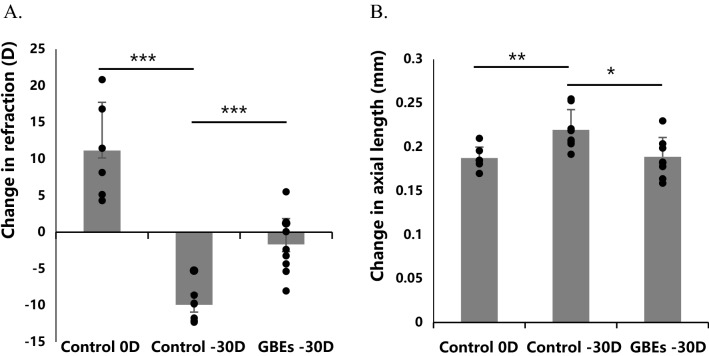
GBEs significantly suppressed a myopic refractive shift and axial elongation in a murine LIM model. (**A**). In the control 0 D and − 30 D groups, the eyes treated with − 30 D lenses had a significantly greater refractive shift than the eyes treated with 0 D lenses (*p* < 0.001). When compared to the control − 30 D group, mice in the GBEs − 30 D group showed a significantly reduced refractive shift (*p* < 0.001) (n = 8). (**B**). Compared to the control 0 D group, the control − 30 D group showed a significant axial elongation (*p* < 0.01). Compared to the control − 30 D group, the GBEs − 30 D group showed a significantly smaller axial elongation (*p* < 0.05) (n = 8). *p < 0.05, **p < 0.01, ***p < 0.001, bars represent mean + / − standard deviations. The one-way ANOVA was used to determine significant differences.

The effect of oral GBEs administration on the progression of myopia was examined in a murine model of lens-induced myopia (LIM). The model is roughly the same as the previously established^40^, except that the monocular induction of myopia is changed to the simultaneous induction of myopia in both eyes, which is more consistent with the arrangement of our experiment. The concentration of GBEs we selected for oral administration was 0.0667% (200 mg/kg/day), which was the highest concentration without causing hepatotoxic reactions in mice. In this study, only the highest dose of GBEs within the safe range was used to investigate whether it has an inhibitory effect on myopia. Mice were divided into three groups based on lenses and diet, normal chow with 0 diopters (D) lenses group (the control 0 D group), normal chow with − 30 D lenses group (the control − 30 D group), and 0.0667% GBEs mixed chow with − 30 D lenses group (the GBEs − 30 D group), respectively (n = 8 for each group). In the two groups that were fed with normal chow, eyes treated with a − 30 D lens had a significantly higher refractive shift than eyes treated with 0 D (− 9.92 ± 1.53 D vs. + 11.14 ± 6.60 D, *p* < 0.001) (Fig. 2A). When compared to the normal chow group with a − 30 D lens, mice given GBEs mixed chow exhibited a significantly less refractive change with − 30 D lens (− 9.92 ± 1.53 D vs. − 1.67 ± 3.51 D, *p* < 0.001) (Fig. 2A). The axial length changes were as follows, the eyes treated with − 30 D lens showed a significant axial elongation compared with the eyes treated with 0 D lens in normal chow groups (0.22 ± 0.02 mm vs. 0.19 ± 0.01 mm, *p* < 0.01) (Fig. 2B). In the eyes with − 30 D lenses, the GBEs − 30 D group showed a significantly smaller axial elongation compared to the control − 30 D group (0.19 ± 0.02 mm vs. 0.22 ± 0.02 mm, *p* < 0.05) (Fig. 2B). These results suggest that in a murine LIM model, oral GBEs administration reduced a refractive shift and axial elongation. Moreover, we also measured the change in the sum of corneal thickness and anterior chamber depth and the change in lens thickness in the three groups of mice, and no significant changes were observed (Supplementary Fig. 1). Multiple studies have shown that axial length is particularly related to choroidal thickness in myopia^41–44^, Thus we consider that the axial length elongation induced by myopia also brings with it the associated choroidal response.

### Oral GBEs administration increased choroidal blood perfusion in mice

**Figure 3:**
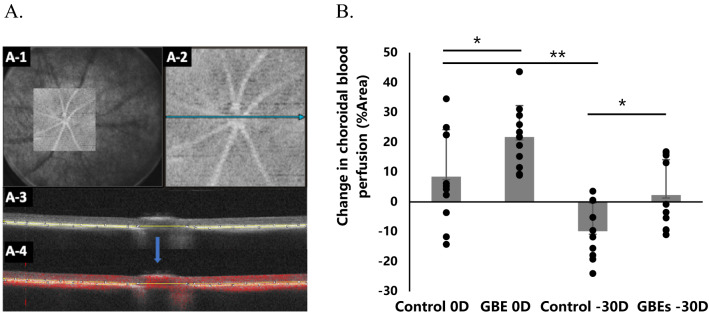
GBEs increased choroidal blood perfusion in a murine model. (**A**). Choroidal blood perfusion was measured by OCTA, which allows for a choroid enface angiogram and corresponding B-scan. (**A**-1). En-face angiography image. (**A**-2). Angiography of 9 mm in length and width centered on the optic nerve. (**A**-3). The En face angiogram corresponds to the B-scan image. The choroid region is circled in yellow on the B-scan image. (**A**-4). OCTA generated a B-scan angiogram of the choroid layer. The proportion of the red dots in the choroid region was calculated using the area measuring tool in ImageJ, the area beyond the red dot (100-%Area) is the choroidal blood perfusion area. (**B**). The changes in choroidal blood perfusion after 3 weeks of GBEs feeding were significantly higher than those in the normal feeding group, regardless of whether myopia induction was performed (*p* < 0.05) (n = 10). *p < 0.05, **p < 0.01, bars represent mean + / − standard deviations. The one-way ANOVA was used to determine significant differences.

**Figure 4:**
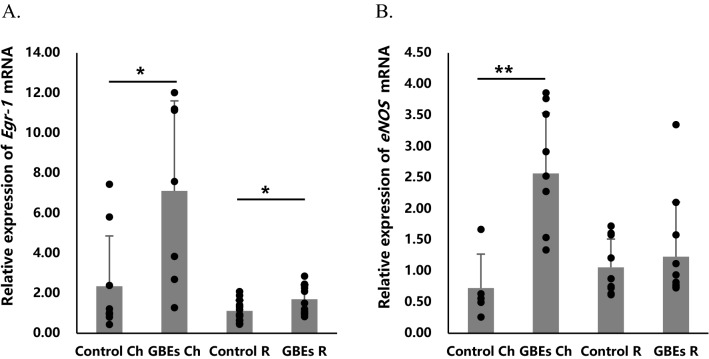
The mRNA expression of *Egr-1* and *eNOS* was significantly upregulated in the choroid by GBEs administration. (**A**) *Egr-1* mRNA expression showed a significant increase in the choroidal and retinal samples after 3 weeks of oral GBEs administration, compared to the normal chow-fed control group (*p* < 0.05) (n = 8). (**B**) *eNOS* mRNA expression also showed a significant increase in the choroid after 3 weeks of GBEs feeding compared to the control group (*p* < 0.01) (n = 8). *p < 0.05, **p < 0.01, bars represent mean + / − standard deviations. Ch: choroid; R: Retina; GBEs: Gingko biloba extracts.

To demonstrate the suppressive mechanism of myopia progression, the mice were randomly divided into 4 groups: 0D lenses with normal diet group (Control 0D), 0D lenses with GBEs diet group (GBEs 0D), binocular myopia induced with normal diet group (Control − 30D), and binocular myopia induced with GBEs diet group (GBEs − 30D) (n = 10 for each group). Choroidal blood perfusion was measured by optical coherence tomography angiography (OCTA), which can provide En face and B-scan images of the choroidal vascular systems. In the overall En face image (Fig. 3A-1), a square frame with a size of 9 mm (width) $\times$ 9 mm (length) is selected for OCTA photography to obtain a partial En face image centered on the optic nerve (Fig. 3A-2). B-scan image corresponding to horizontal lines passing through the optic nerve center in En face angiogram was analyzed as a unified standard. In the relative B-scan image, the choroid is represented by the white highlighted signal area in the yellow coil (Fig. 3A-3). With the addition of angiography, the red dots represent the signals without blood perfusion, while the area beyond the red dots represents the area through which blood perfusion passes (Fig. 3A-4). The proportion of red pixels on the choroid region was measured by ImageJ, and the choroidal blood perfusion was calculated by scoring the percentage of non-red pixels on the total choroid region. In the histogram of the change in choroidal blood perfusion, compared with the control 0D group, mice in the GBEs 0D group showed a significantly greater change in blood perfusion (8.48 ± 15.75%Area vs. 21.74 ± 10.54%Area, *p* < 0.05) after 3 weeks of feeding, meanwhile, mice in the Control − 30D group showed a decrease in choroidal blood perfusion (8.48 ± 15.75%Area vs. − 9.82 ± 9.47%Area, *p* < 0.01). In the case of myopia induction, choroidal blood perfusion was improved in the GBEs − 30D group, compared to the control − 30D group (2.29 ± 11.84%Area vs. − 9.82 ± 9.47%Area, *p* < 0.05) (Fig. 3B).

### GBEs increased a significant ***Egr-1*** and ***eNOS*** expression in real-time PCR

To further verify the mechanism of GBEs in inhibiting the progression of myopia, we collected the choroidal and retinal samples from the mice in the Control 0D group and the GBEs 0D group for real-time PCR (n = 8 for each group). The results showed a significantly increased *Egr-1* mRNA expression in the choroid after 3 weeks of GBEs feeding compared to the control group fed with normal chow for 3 weeks (7.11 ± 4.5 folds vs. 2.34 ± 2.52 folds, *p* < 0.05). In the retina, *Egr-1* mRNA expression was significantly upregulated after 3 weeks of GBEs feeding compared with the control group (1.69 ± 0.73 folds vs. 1.11 ± 0.53 folds, *p* < 0.05) (Fig. 4A). In addition, *eNOS* mRNA expression was significantly upregulated in the choroid after 3 weeks of GBEs feeding compared with the control group (2.56 ± 0.97 folds vs. 0.72 ± 0.55 folds, *p* < 0.01) (Fig. 4B).

### GBEs inhibited choroidal thinning in a murine LIM model

**Figure 5:**
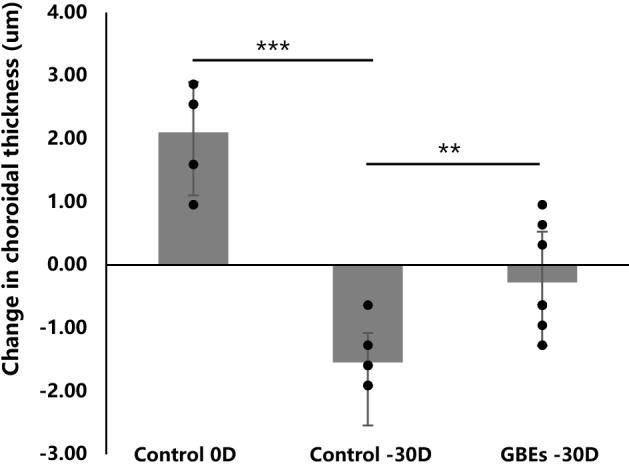
GBEs administration reduced choroidal thinning. The two groups that were given normal chow showed a significant difference in choroidal thickness between the − 30 D lenses group and the 0 D lenses group (*p* < 0.001). Mice given GBEs chow with − 30 D lenses showed a statistically significant increase in choroidal thickness compared to the normal chow group with − 30 D lenses (*p* < 0.01) (n = 6). **p < 0.01, ***p < 0.001, Bars represent mean + / − standard deviations. The one-way ANOVA was used for statistical analysis. Control 0 D: mice fed with normal chow with 0 D lenses in both eyes; control − 30 D: mice fed with normal chow with − 30 D lenses in both eyes; GBEs − 30 D: mice fed with GBEs with − 30 D lenses in both eyes.

The above experiments have verified that GBEs administration can increase the amount of blood perfusion in the choroid regardless of whether it was accompanied by myopia induction. Subsequently, to explore the influence of changes in choroidal blood perfusion caused by GBEs administration on choroidal thickness, mice wearing 0D lenses were fed with normal chow and 0.0667% GBEs chow respectively, and choroid thickness was measured after 3 weeks of feeding, we found that choroidal thickness significant thicken in 0.0667% GBEs chow group (Supplementary Fig. 2). Whereafter, myopia was induced in 3-week-old mice and fed with normal chow or 0.0667% GBEs mixed chow until 6 weeks of age, the changes of choroidal thickness before and after feeding were compared between groups (n = 6 for each group). In the control − 30 D group, a significant decrease in choroidal thickness was demonstrated compared to the control 0 D group (− 1.55 ± 0.47 um vs. + 2.10 ± 0.80 um, *p* < 0.001) (Fig. 5). When compared to the control − 30 D group, mice in the GBEs − 30 D group exhibited a significantly less choroidal thickness change (− 1.55 ± 0.47 um vs. − 0.28 ± 0.81 um, *p* < 0.01) (Fig. 5). Those findings further corroborate existing evidence that the change in choroidal blood perfusion was positively correlated with the change in choroidal thickness in a murine LIM model.

## Discussion

In this study, the activation of GBEs in vitro for EGR-1 using luciferase assay was measured and the dose-dependent activation of GBEs on EGR-1 was confirmed. Following this experiment, the suppressive effect of oral GBEs administration on myopia progression was investigated using a murine LIM model and it was found that GBEs oral administration may inhibit changes in refraction and axial length. Subsequently, under the condition of myopia induction and no myopia induction, comparing the changes in choroidal blood perfusion in mice given GBEs orally with mice not given, it was found that oral GBEs administration significantly increase choroidal blood perfusion in mice with or without myopic induction. In addition, a significant upregulation of *Egr-1* and *eNOS* mRNA after oral GBEs was also confirmed using real-time PCR. The decreases in choroidal thickness were mitigated after oral administration of GBEs in a murine LIM model.

The main category of GBEs active ingredients are the ginkgolides, the bilobalides (also known as terpenes), and the flavonoids^45,46^. Flavonoids have the ability to dilate blood arteries by boosting the release of endothelial-derived relaxing factors and prostacyclin^47^. Early studies have noted the influence of GBEs on blood flow. Oral administration of GBEs has been proven in animal models to greatly relieve cerebral and myocardial ischemia, as well as reduce subsequent ischemic damage^48–50^. In the application of ocular diseases, after GBEs oral administration, the end-diastolic velocity in the ophthalmic artery and the ocular blood flow increased, which has a great therapeutic potential for some ischemic eye diseases and is currently being used as a potential treatment option for normal-tension glaucoma^35,51,52^. In our research, OCTA was used for the first time in a mouse model to evaluate the changes in choroidal blood perfusion with and without GBEs oral administration. Prior to our study, there had been studies that used OCTA to measure choroidal blood perfusion in guinea pig myopic models^38,39,53^. These studies provide strong technical support for the application of OCTA in mouse models. The OCTA can reduce image noises in a matter of seconds, increasing the detail, and improving visibility and the accuracy of choroid blood perfusion measurement^54^. Our experiment intuitively demonstrated that with or without myopic induction, increased choroidal blood perfusion was detected after 3 weeks of GBEs feeding. and combined the characteristics of GBEs in improving choroidal blood perfusion with the inhibition of myopia progression, which provides evidence of the association between myopia and choroidal blood perfusion.

To verify the possible mechanism of suppressing the progression of myopia by GBEs administration, we used real-time PCR to detect the expressions of *Egr-1* and *eNOS* in the retina and the choroid after three weeks of GBEs oral administration. The rationale for observing *eNOS* expression is that previous studies have revealed that *eNOS* regulates vascular tone and angiogenesis, and its expression was elevated in arteries subjected to higher blood flow^55,56^. Consistent with the results in vitro experiment, after oral administration of GBEs, *Egr-1* expression was significantly upregulated in both the choroid and the retina. For the expression of *eNOS*, the choroid showed a considerable upregulation of *eNOS* expression, whereas the retina showed a tendency but not a statistically significant rise. These results correspond to an increase in choroidal blood perfusion as evaluated by OCTA. Therefore, we consider that there may be two different mechanisms by which GBEs suppress the progression of myopia. First, it suppresses axial eye growth by upregulating the expression of *Egr-1* in the choroid and the retina. Furthermore, speculate from our experimental results, GBEs administration induce the upregulation of the *eNOS* expression in the choroid, which can relax vascular smooth muscle cells, expands choroidal vascular vessels, increases choroidal blood perfusion, and maintains the thickness of the choroid and the position of the retina, leading to suppression of the development of myopia. In addition, some studies have also shown that treatment with GBEs increases dopamine release in the rat medial prefrontal cortex^57,58^. Dopamine, as a major neurotransmitter of the retina, promotes its release and can inhibit the development of myopia^59^. It is inferred that another mechanism by which GBEs inhibit the progression of myopia may be that it induces the release of dopamine in the retina, which needs to be verified by further experiments.

However, this study has some limitations. Only a concentration of 0.0667% of GBEs was selected to verify its suppressive effect on the progression of myopia. The selection of oral administration concentration of GBEs was summarized from several reports of GBEs pharmacological tests on mice. Oral dosages from 100 to 400 mg/kg have been reported, and GBEs dosages of more than 200 mg/kg have been found to cause hepatotoxicity in rodents^60–64^. In this experiment, we made 0.0667% GBEs containing chow, which corresponds to 200 mg/kg, to verify its suppressive effect on the progression of myopia. Since the drug concentration used was considerably high, further studies are needed to set several groups of different concentrations to better understand the optimal concentration of GBEs oral administration to suppress the progression of myopia.

The prevalence of myopia is increasing rapidly and continuously all over the world, it is urgent to explore and develop methods to strategies for controlling myopia in children. Until now, our research is the first to demonstrate how GBEs, a natural extract, effectively control myopia in mice. As preliminary research based on clinical trials, our research laid the foundation for the research of GBEs administration suppressing myopia progression and provides the possibility for subsequent dose-related experiments. For humans, GBEs is generally well-tolerated and harmless but also can cause some side effects. It has been reported that the maximum recommended dose for GBEs is 240 mg/day for adults^65^, but there have been no definitive studies on recommended doses for children. For future prospective clinical studies, how to determine the safe dose and fully exploit the impact of GBEs on myopia inhibition in children are topics that require extensive discussion.

## Materials and methods

The Institutional Animal Care and Use Committee at Keio University.authorized all operations (approval number: 16017). All methods and experimental protocols followed the National Institutes of Health (NIH) guidelines for working with laboratory animals and the ARVO Animal Statement for the Use of Animals in Ophthalmic and Vision Research. Our study was also following ARRIVE guidelines and adhered to the random assignment principle.

### Establishment of EGR-1 luciferase permanent expression cell line

The 293AAV Cell Line is derived from the parental 293 cell line and developed by Cell Biolabs, Inc. after cloning and multiple rounds of testing (Cell Biolabs, Inc. California, USA cat#AAV-100). HEK 293AAV cell line purchased from the company was transfected with Egr1-luciferase reporter gene construct (Cignal Lenti EGR1 Reporter (Luc), Qiagen, Venlo, Netherlands) to monitor EGR-1 transcriptional activity. The method followed the way previously reported^22^. Briefly, Cignal Lenti Egr-1 reporter Luc (QIAGEN, Netherland #CLS-5021L) virus was used to infect cells for less than 20 h using SureENTRY Transduction reagent (QIAGEN, Netherland #336,921). (25 MOI). After infection, cells were passaged once and then grown until no infected cells died entirely. Five colonies were picked up and seeded in 2.0 × 10^4^ cells/well in the HTS Transwell®-96 Receiver Plate. Among all the cell lines transfected with Egr-1-luciferase reporter gene construct purchased by our laboratory, the HEK 293AAV EGR-1-Luc cell line was the ablest to stably express EGR1 activity after PMA (positive control) treatment, thus, this cell line was chosen as the most reactive colony for the screening assay.

### Luciferase assay

Luciferase assay was performed as reported previously^22^. The HEK 293AAV EGR-1 cells that were passaged twice were transferred to HTS Transwell®-96 Receiver Plate, White, TC-Treated, Sterile (Corning, New York, USA #3783), added 70ul medium contained with 10%FBS to each well and the number of cells per well was 2.0 × 10^4^, incubated overnight at 37 °C in a 5% CO_2_ incubator. GBEs (INDENA JAPAN CO., Tokyo, Japan #9,033,008) was weighed and dissolved in DMSO (Wako, Tokyo, Japan #043-07,216) to form a 100 mg/ml solution and let stand away from light overnight at room temperature after agitation. Dissolve the supernatant in DMSO at a ratio of 1:400, 2:400, 3:400, so that the final concentration becomes 0.25 mg/ml, 0.50 mg/ml and 0.75 mg/ml, added them at 70ul to each well. Cells were incubated for 18 h at 37 °C in a 5% CO2 incubator.

Positive control of 100 nM PMA (Promega, USA #V1171 or Abcam, England #16,561-29-8) was employed, which is known as an EGR-1 activator^66^. DMSO-containing media without any chemicals was used as negative control. The concentration of GBEs we used was 0.25 mg/ml, 0.50 mg/ml, and 0.75 mg/ml, respectively. First of all, in prior studies, we found that 0.25 mg/ml of crocetin was the maximum concentration soluble in DMSO, which induced a significant Egr-1 activation in a luciferase assay^22^. Based on this experience, we configured 0.25 mg/ml of GBEs as the preferred concentration. Secondly, for mice, several studies have confirmed that GBEs feeding more than a daily dose of 200 mg/kg can cause hepatotoxicity in rodents^62–64^. After concentration conversion, the in vivo experimental concentration corresponding to the feeding concentration of 200 mg/kg/day is 0.50 mg/ml. Therefore, we set 0.5 mg/ml GBEs as the second concentration. Finally, 0.75 mg/ml GBEs was set as the third concentration to explore the change of EGR-1 activity under ultra-high-concentration conditions in vitro. The ONE-GloTM Luciferase Assay System (Promega, Madison, Wisconsin, USA #E6110) was used to quantify luciferase expression. After evenly mixing One 10 ml Glo™ Luciferase Assay Buffer and 1 vial ONE-Glo™ Luciferase Assay Substrate of ONE-Glo™ Luciferase Assay System, add 70ul for each well. We identify fluorescence intensity at Synergy HTX (Biotek, Vermont, USA) under the following conditions: shaking linear for 30 s, delay for 12 min, gain 180, integration time 0.5 s, and reading height 1.0 mm.

### Mouse model

C57BL6/J mice (CLEA, Shizuoka, Japan) were kept in conventional transparent mouse cages (29 × 18 × 13 cm) of four or five per cage in an air-conditioned room maintained at 23 ± 3 °C with a 12-h diurnal period and unrestricted access to normal food (MF, Oriental Yeast Co., Ltd, Tokyo, Japan) and tap water. For GBEs-fed mice, as the average food intake of a 10 g mouse is about 3 g per day^67,68^, the feeding was carried out according to the approximate weekly food intake per cage we calculated. The mice's weight was recorded weekly to ensure that GBEs feeding did not affect their weight gain compared to normal food intake mice, records showed no significant difference in body weight between the normal feeding and GBEs feeding groups (Supplementary Fig. 3). Change the feed, tap water, and cage once a week to ensure that the living space of the mice is clean. For anesthesia, the dose of anesthesia was adjusted for each mouse's body weight, ensuring that approximately 0.1 ml/10 g of anesthesia was administered. To minimize errors, all experiments used mice of the same sex (male) and weight (10 ± 1 g), and all mice were randomly assigned. To ensure the uniformity of the experiment and minimize the influence of objective factors on the experimental results, during the whole experiment, all the mice were kept under uniform light and temperature conditions, ensuring that the time of each experiment measurement is consistent, and the average measurement time of each mouse was tried to be the same.

### Analysis of dietary factors in the LIM model

A murine LIM model was prepared as previously reported^40^. We created a mouse eyeglass frame that conformed to the contour of the mouse's head and printed it out using a three-dimensional printer. A negative 30 D lens made of PMMA was created for myopia induction. Myopic induction using the − 30 D lens showed greater myopic shift compared to the form-deprivation myopic model^40^. With some differences from the LIM model used previously, we used binocular myopic induction instead of monocular induction. The left and right eyes of the glasses were adjusted by the shape of the mouse skull frame and fixed on the stick with a screw, and then glued the Stick to the mouse skull with a self-cure dental adhesive system. This was done under general anesthesia with the combination of midazolam (Sandoz K.K., Minato, Japan), medetomidine (Domitor®, Orion Corporation, Turku, Finland), and butorphanol tartrate (Meiji Seika Pharma Co., Ltd., Tokyo, Japan) (MMB). The dosage for each mouse was 0.01 ml/g.

During the myopia induction phase, mice were given either normal (MF, Oriental Yeast Co., Ltd, Tokyo, Japan) or mixed chow containing the candidate chemical 0.0667 percent GBEs (INDENA JAPAN CO., Tokyo, Japan #9,033,008). 0.0667% GBEs contain 24% of the flavonol glycosides of quercetin, kaempferol, and isorhamnetin and 6% terpene trilactones. The corresponding concentration of GBEs mixed chow was 200 mg/kg/day, which is consistent with the concentration of GBEs that causes the significantly high activity of EGR-1 in vitro experiments. The addition of GBEs and the production of 0.0667% GBEs mixed chow are all produced by chow manufacturing company (Oriental Yeast Co., LTD., Tokyo, Japan).

### Measurement of refraction, axial length, and choroidal thickness by infrared photorefractor and SD-OCT

At the beginning (3-week-old) and the conclusion (6-week-old) stage of the myopia induction, refraction, axial length and choroidal thickness were measured using an infrared photorefractor (Steinbeis Transfer Center, Stuttgart, Baden-Württemberg, Germany) and an SD-OCT system (Envisu R4310, Leica, Microsystems, Wetzlar, Germany). All measurements were carried out with mydriasis eye drops containing 0.5% tropicamide and 0.5% phenylephrine (Santen Pharmaceutical Co., Ltd, Osaka, Japan) and all were performed under MMB general anesthesia. The refractive values were measured with a continuous data trail. Along with the corneal vertex reflection, the axial length was measured from the anterior corneal surface to the retinal pigment epithelium^40^. The choroidal thickness was measured according to the previous report^22,69^. Briefly, the mean choroidal thickness was computed by the choroidal area distant from the disc, at the border of the retinal pigment epithelium and the posterior surface of the choroid, the radius at 0.5 mm from the ringed disc was obtained by ImageJ quantitative analysis (National Institutes of Health, Bethesda, Bethesda, Maryland, USA). The average choroid thickness was then determined by dividing the area by the circumference.

### Dietary effect on refraction, axial length, choroidal thickness, and choroidal blood perfusion

To compare the effects of dietary factors on refraction, axial length, and choroidal thickness in mice, 3-week-old C57BL6/J mice were randomly divided into the control 0 D group, the control − 30 D group, and the GBEs − 30 D group. The control 0 D group and the control − 30 D group were fed with normal chow and wore 0 D lenses and − 30 D lenses on both eyes respectively. The GBEs − 30 D group was fed with 0.0667% GBEs mixed chow while wearing − 30 D lenses on both eyes. During this period, myopia induction was carried out simultaneously. Both feeding and myopic induction were performed from 3 to 6 weeks of age. Since we used binocular myopia induction instead of monocular myopia induction, the data from each eye was analyzed independently. To compare the effects of dietary factors on choroidal blood perfusion in mice, 3-week-old C57BL6/J mice were randomly divided into 4 groups, two groups were given a normal diet and a GBEs diet without myopic induction, and the other two groups were given a normal diet and a GBEs diet while myopic induction was performed. They were fed from 3 to 6 weeks of age.

### Analysis of choroidal blood perfusion

Choroidal blood perfusion was measured at the initial stage (3-week-old) and the end (6-week-old) stage using an SS-OCT / OCTA device (XEPHILIO OCT-S1, CANON Medical Systems, Tokyo, Japan), which adopts swept-source technology to allow the light source to reach deep in the fundus, and a wide range from vitreous to retina, choroid, and sclera boundary can be imaged in high definition. OCTA determines blood flow in vivo by analyzing variations in intensity and phase information from the mobility of red blood cells observed by repeated OCT scans at the same location^70,71^. A square area of 9 mm (width) $\times$ 9 mm (length) centered on the optic nerve was selected to construct En face angiogram, while 464 consecutive OCTA B-scans at the level of the full retina-choroid-sclera were obtained at each recording position to observe the changes of blood perfusion signal at different positions. Since each angiography has a matching B-scan image, in our research, B-scan images corresponding to En face angiography across the central region of the optic nerve were analyzed as a unified standard. In the B-scan image, we observed that there was no red dot covering the choroid vessels, and the results obtained after calculating the area not covered by red dots as choroid blood perfusion were consistent with the existing research results, that is, choroid blood perfusion decreased with the induction of myopia^38^. Thus, we considered that for the C57BL6/J mice, the blood perfusion signal is reversed below the RPE due to the abundant pigment particles in the RPE layer. The area without blood perfusion is covered by red noise points, which are also known as flow voids (FVs), and some studies have found that choroidal blood perfusion was negatively correlated with the FVs of choriocapillaris^72,73^. In this study, we used the polygon selections tool of ImageJ to circle the entire choroid region in the B-scan image and analyze the area ratio of FVs in the whole choroid region, which was an algorithmic threshold selection used to calculate the proportion of red pixels. Choroidal blood perfusion was calculated by scoring the percentage of non-red pixels about the number of total pixels.

### Real-time PCR analysis

After choroidal blood perfusion was assessed by OCTA, mice were injected with an overdose of MMB to produce profound anesthesia and euthanized through cervical dislocation. Subsequently, eyeballs were enucleated to separate choroidal and retinal tissues which were immediately frozen in liquid nitrogen and kept at − 80 °C.

For real-time PCR, tissues were lysed in TRI reagent (MOR, Miami, FL, USA #TR118) to solubilize denatured proteins and separate tissue RNA. Add RWT (QIAGEN, Hilden, Germany, #1,067,933) and RPE (QIAGEN, Hilden, Germany, #1,018,013), which were dissolved in EtOH in a ratio of 1:2 and 1:4 to isolate small RNAs and remove the traces of salts. RNA samples were dissolved in Rnase-free water (TAKARA HOLDINGS INC., Kyoto, Japan, 9012) and measured with a spectrophotometer (NanoDrop; ThermoFisher Scientific, Waltham, MA, USA) for gene expression analysis.

The extract RNA (200 ng) was converted to cDNA using 4xDN Master Mix with gDNA remover (TOYOBO, Osaka, Japan, #FSQ-301), 5xRT Master MixII.SYBR green RT-PCR was performed using THUNDERBIRD SYBR qPCR Mix (TOYOBO, Osaka, Japan, #QPS-201), and PCR was performed using StepOnePlus Real-Time PCR System (Applied Biosystems, Waltham, Massachusetts, USA). The 2 − ΔΔCt method was used to quantify differential gene expression, which was then standardized to the reference gene (*GAPDH*). The primer sequences for qPCR were as follows:mouse *Egr-1* forward: CCACAACAACAGGGAGACCT,mouse *Egr-1* reverse: ACTGAGTGGCGAAGGCTTTA,mouse *eNOS* forward: TCCGGAAGGCGTTTGATCmouse *eNOS* reverse: GCCAAATGTGCTGGTCACCmouse *GAPDH* forward: AGGAGCGAGACCCCACTAACmouse *GAPDH* reverse: GATGACCCTTTTGGCTCCAC

### Statistical analysis

All results are expressed as mean ± standard deviation (SD) and analyzed using a blind procedure. An independent *t*-test or one-way ANOVA was used to assess the statistical significance of the differences (Microsoft Excel 2003, USA), and results with *p*-values < 0.05 were considered significant. Power analysis was performed on the Cancer Research Network (SWOG), used for results with a *p*-value < 0.05, the calculated power is all greater than 0.8.

## Supplementary Information

Supplementary Figures.

Supplementary Information 2.

## Data Availability

All data generated or analysed during this study are included in this published article and its supplementary information files.
