# Supplementary material for: Ginkgo biloba extracts improve choroidal circulation leading to suppression of myopia in mice
Source: Sci Rep. 2023 Mar 7;13:3772. doi: 10.1038/s41598-023-30908-1 (PMC9989591; doi:10.1038/s41598-023-30908-1)
Supplement: Supplementary file 1 — Supplementary Figures. [file 41598_2023_30908_MOESM1_ESM.docx]

**Supplementary** **Materials for**

**Ginkgo Biloba Extracts Improve Choroidal Circulation Leading to Suppression of Myopia in Mice**

Jing Hou^1,2*^, Kiwako Mori^1,2*^, Shin-ichi Ikeda^1,2^, Heonuk Jeong^1,2^, Hidemasa Torii^1,2^, Kazuno Negishi^1^, Toshihide Kurihara^1,2*^, Kazuo Tsubota^1,3*^

**The number of supplementary figures: 3**

**Supplementary Figure 1:**

A. B. C.


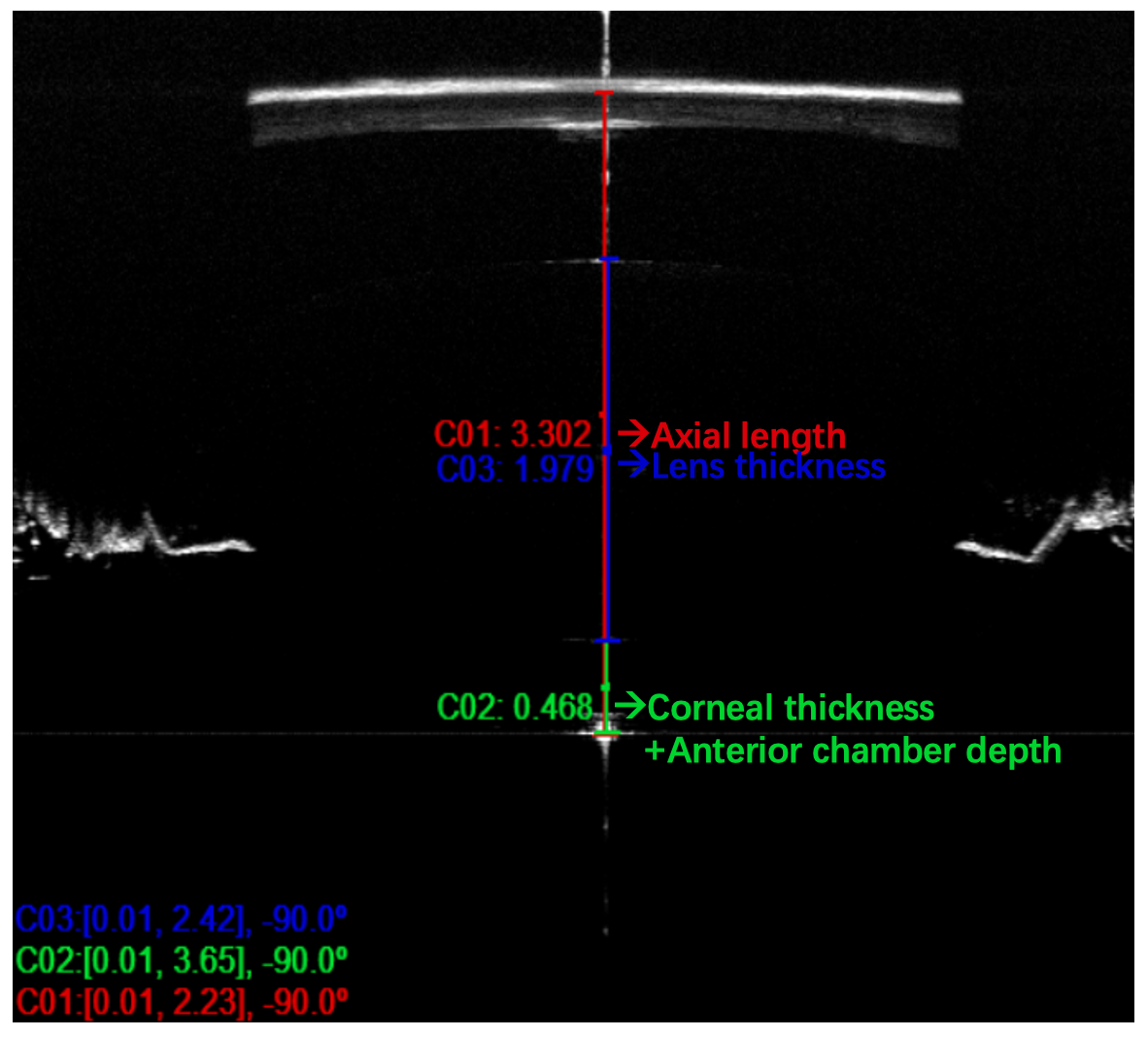

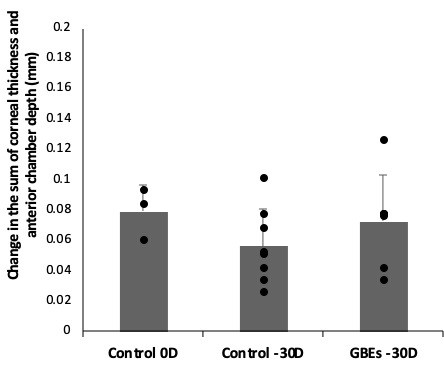

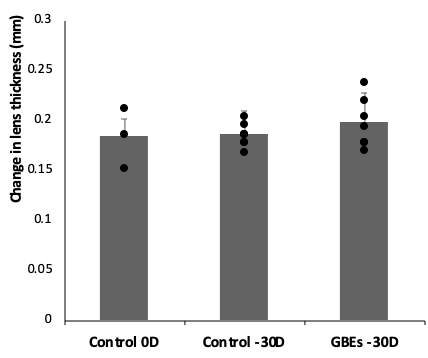


**Supplementary Figure 1:** **Change of ocular parameters after 3 weeks of myopic induction or combined GBEs feeding.** A. The ocular parameters were measured using SD-OCT. We defined the AL as the length between the corneal vertex and the retina's outer boundary and measured the sum of corneal thickness, anterior chamber depth, and lens thickness. B. Change in the sum of corneal thickness and anterior chamber depth after 3 weeks of 0D, and −30D lens wearing, and 0.0667% GBEs mixed chow with −30 D lens wearing started from p21. No significant change was observed (n=8). C. Change in lens thickness also showed no significant change between the control 0D group, the control -30D group, and GBEs -30D group (n=8). bars represent mean +/− standard deviations.

**Supplementary Figure 2:**

**Supplementary Figure 2: GBEs administration can improve choroidal thickness in 0D lens-treated mice.** Compare with normal chow with 0D lens group, mice fed with GBEs mixed chow showed a significantly greater change in choroidal thickness after 3 weeks of feeding (n=14, respectively). **p < 0.01.*t*-test. Bars represent mean +/− standard deviations.

**Supplementary Figure 3:**

**Supplementary Figure 3: Change in body weight after 3 weeks of GBEs feeding or myopia induction.** After 3 weeks of GBEs feeding or myopia induction, there was no significant difference in body weight change between the 4 groups (n=8, respectively). One-way ANOVA. Bars represent mean +/− standard deviations.
